# Tailoring HIV Care for Black Populations: A Pilot Feasibility Prospective Cohort Study

**DOI:** 10.2196/56411

**Published:** 2024-10-04

**Authors:** BreAnne Young, Olveen Carrasquillo, Deborah L Jones, Yue Pan, Sonjia Kenya

**Affiliations:** 1 Department of Medicine University of Miami Miller School of Medicine Miami, FL United States; 2 Clinical and Translational Science Institute University of Miami Miller School of Medicine Miami, FL United States; 3 Department of Psychiatry and Behavioral Sciences University of Miami Miller School of Medicine Miami, FL United States; 4 Department of Public Health Sciences University of Miami Miller School of Medicine Miami, FL United States

**Keywords:** community health workers, CHWs, society, social determinants of health, social factor, social disparity, social inequality, social inequity, HIV disparities, HIV, human immunodeficiency virus, AIDS, feasibility, care continuum, Black, African American

## Abstract

**Background:**

Research has shown that integrating community health workers (CHWs) into the formal health care system can improve outcomes for people living with HIV, yet there is limited literature exploring this framework among marginalized minority populations.

**Objective:**

Herein, we discuss the feasibility of a clinic-embedded CHW strategy to improve antiretroviral therapy adherence among Black people living with HIV in Miami-Dade County, Florida, a designated priority region for the US Department of Health and Human Services’ Ending the HIV Epidemic Initiative.

**Methods:**

From December 2022 to September 2023, three CHWs were trained and integrated into the hospital workflow to provide support as members of the clinical team. Ten Black adults with an HIV viral load over 200 copies/mL were enrolled to received 3 months of CHW support focused on navigating the health system and addressing poor social determinants of health. Intervention feasibility was based on 4 criteria: recruitment rate, demographic composition, study fidelity, and qualitative feedback on CHW perceptions.

**Results:**

Participants were recruited at a rate of 5.7 participants per month, with the sample evenly distributed between men and women. Retention was moderately strong, with 7 (70%) of the 10 participants attending more than 75% of CHW sessions. Qualitative feedback reflected CHW perceptions on clinical interactions and intervention length.

**Conclusions:**

Outcomes indicate that a clinic-integrated CHW approach is a feasible and acceptable methodology to address adverse social determinants and improve HIV treatment adherence. By offering targeted social and clinical support, CHWs may be a promising solution to achieve sustained viral suppression and care engagement for Black people living with HIV.

## Introduction

Advances in antiretroviral therapies (ARTs) have led to significant improvements in HIV treatment and management, transforming the disease from a fatal diagnosis to a manageable chronic illness. Despite this, Black populations are disproportionately impacted by poor HIV outcomes, and the incidence rate among the Black communities in Miami-Dade County, Florida, is among the highest in the United States. Accounting for 15% of the region’s population, Black Miamians comprise nearly 70% of the region’s AIDS-related deaths, highlighting vast underutilization of HIV resources [1,2].

The complex determinants of health and health behaviors that contribute to HIV disparities stem from systemic discrimination that has historically limited access to services and diminished the continuity of care for marginalized populations [3]. The legacy of such discriminatory care continues to impact these communities, as those who were barred from equitable health services are often the ones who are most affected by poor HIV outcomes [4]. As innovative HIV treatment and prevention modalities continue to emerge, underutilization of such advances by Black Miamians have become even more prominent, highlighting the need for evidence-based HIV interventions that are tailored to reach those who are most affected by HIV.

Community health worker (CHW) interventions have emerged as an efficacious strategy to improve HIV outcomes in Black populations [5]. As trusted members of the community, CHWs have the capacity to deliver patient-centered care using culturally adapted strategies that are tailored to the populations they serve [6]. As the interface to the formal health care system in highly vulnerable communities, CHWs facilitate collaboration among health care providers, patients, and social supports, such as family and friends—an integral factor in improving health and promoting wellness among people living with HIV. Further, by focusing on the delivery of HIV services that “meet people where they are,” CHWs are able to facilitate the communities of greatest need in navigating the social and structural barriers to engagement in care, such as stable housing, food security, drug assistance, and immigration status.

For example, CHWs in our formative studies assessed risk behaviors; provided peer education on treatment adherence; and navigated patients to the appropriate social support services, including food banks, cultural groups, churches, and HIV support groups. Functioning as peers, HIV educators, and liaisons to the health care system, CHWs provided Black people living with HIV with the multifaceted support needed to improve medication adherence and significantly reduce their HIV viral loads.

Building upon the CHW framework, research suggests that integrating CHWs into the formal health care system could further improve access to treatment and preventive services for people living with HIV [7,8]. However, there is limited literature on clinic-integrated CHW models for minority populations, and less so on their unique consideration of the cultural nuances and social barriers that diminish access to care among marginalized, racial minority communities [9,10]. To this end, we developed the Integrated Navigation and Support for Treatment, Adherence, Counseling, and Research (INSTACARE) intervention: a CHW strategy to improve ART adherence and reduce HIV viral loads among Black people living with HIV. Herein, we discuss the design and examine the feasibility, fidelity, and acceptability of the intervention within a clinical setting and the potential impacts of the intervention in achieving the identified objectives.

## Methods

### Overview

From December 2022 to May 2023, we conducted a pilot, feasibility, prospective cohort study aimed at determining the fidelity, acceptability, and potential impact of a CHW intervention for Black adults in care at an outpatient HIV clinic at a large safety-net hospital in Miami-Dade County [11].

### Ethical Considerations

Prior to study onset, this research was reviewed and approved by the University of Miami’s Institutional Review Board (20201234). Participants were verbally informed of the study purpose, procedures, risks, and benefits prior to enrollment. All participants completed written informed consent and were provided a physical copy of the consent form upon request. Participants received US $200 cash compensation for their time. Each participant was assigned a unique subject ID upon enrollment to ensure confidentiality, and all data were deidentified prior to analyses.

### Study Design

Utilizing the Social Ecological Model, CHWs delivered 3 months of patient support informed by the formative qualitative research for this study [12]. Specifically, this clinic-integrated intervention focused on challenges to viral suppression and the complex, multilevel barriers to care experienced by Black people living with HIV in Miami, including restrictive HIV policies, logistical hurdles to navigating care, and stigma across clinical and community-based settings.

### CHW Intervention

CHW integration into the Outpatient Clinical Care Team involved CHWs shadowing the HIV providers. Shadowing was an on-site learning process in which the CHWs observed and followed clinic staff during patient interactions to learn about the wide range of medical, mental health, and social support resources available through the clinic. CHWs established a rotating schedule to ensure at least 1 person was stationed in the clinic daily to network with providers, encourage collaboration with clinic staff, and facilitate immediate study eligibility screening for walk-in patients. CHWs also accompanied patients to appointments to facilitate communication between patients and providers, as well as aid in with scheduling appointments and referrals. When available, CHWs also attended case manager (CM) meetings when their patients were being discussed. At these meetings, CHWs helped to inform the health care team of areas of concern and contribute to the development of intervention plans, inclusive of the information CHWs learned through interacting with their patients. These clinical activities were conducted in addition to the home visits and off-hours communication CHWs traditionally provide to their patients as part of their patient-centered approach to care delivery.

For the first 30 days of enrollment, CHWs met with participants on a weekly basis to discuss their challenges and develop an action plan on how to address those barriers. For example, if employment was the concern, CHWs would offer to help the participant identify a job placement agency and schedule an interview. After the first 30 days of enrollment, meetings were reduced from weekly to biweekly. Some participants, however, were kept on a weekly meeting schedule depending on the complexity of support needed.

Study team meetings with the CHWs, research associates, and data manger occurred weekly. In team meetings, CHWs were encouraged to share documentation on any barriers they encountered in adhering to the protocol or in securing services and resources for their patients.

### CHW Characteristics

Three CHWs were selected for pilot implementation. All 3 CHWs were Black women born and raised within the high-priority jurisdictions in which our participants reside. One CHW was of Haitian descent and fluent in both English and Creole. All 3 women had prior professional experience in social work, and 2 were considered “senior CHWs” with more than 20 years of experience in HIV care and 15 years of experience as CHWs. All CHWs completed HIV counseling coursework provided by the Florida Department of Health, which introduces learners to standardized information regarding the basics of HIV as it pertains to risk reduction, testing, and linkage. CHWs also completed human subjects research training provided by the Collaborative Institutional Training Initiative, including the development of human subject protections, ethical considerations for vulnerable populations, and current regulatory and guidance information.

### Participant Recruitment

Eligible participants included English-speaking, Black patients 18 years of age or older, living with an unsuppressed HIV viral load, as indicated by more than 200 copies of HIV per mL of blood. From December 2022 to May 2023, participants were recruited through referral by the CM or charge nurse on shift, who would identify eligible participants on the clinic’s appointment schedule. The nurse or CM briefly described the study to potential participants. Those who expressed interest in participating were then referred to a CHW to learn more about the study. CHWs then met potential participants directly in the clinic or called them at a later time to provide additional study details. Permission was given during the informed consent process to allow CHWs to access each patient’s viral load data.

### Feasibility Measures

Intervention feasibility was based on 4 criteria: recruitment rate, demographic composition, study fidelity, and qualitative feedback on CHW perceptions.

#### Recruitment Rate

The participant recruitment rate was calculated by dividing the total number of participants enrolled by the number of months in which recruitment occurred. A target recruitment rate was derived from our prior research studies examining the impact of CHW support on HIV outcomes for people living with HIV. Based on recruitment data from our prior research, a 4-person team of full-time CHWs could recruit approximately 14 participants per month. Scaling this value to align with the 3-person team employed in this study, a recruitment rate of 10 participants per month was selected.

#### Demographic Composition

We sought to recruit an equal distribution of men and women within our eligibility criteria. Characteristics of the sample, including frequencies and descriptive statistics, are displayed in Table 1.

**Table 1:** Baseline characteristics of patients enrolled in the INSTACARE^a^ pilot feasibility study (N=10).

Characteristics			Value
**Sex (female), n (%)**			5 (50)
**Age (years), median, (IQR)**			55 (45-58)
**Ethnicity (Haitian), n (%)**			3 (30)
**HIV viral load (copies/mL), range**			214-343,000
**Primary barrier to care, n (%)**			
	Basic physiological needs	5 (50)	
	Social support	3 (30)	
	Health care navigation	2 (20)	

^a^INSTACARE: Integrated Navigation and Support for Treatment, Adherence, Counseling, and Research.

#### Study Fidelity

Study fidelity, calculated by the number of successful patient visits divided by the number of visits attempted [13], was abstracted from CHW case notes. Literature on acceptable retention rates for pilot interventions suggest that “at least 70% of participants in each arm should attend at least 70% sessions” [14,15]. During the 3-month intervention period, CHWs kept case notes and weekly activity logs to document their patient encounters and attempted visits.

#### CHW Experience

CHWs participated in a semistructured focus group at the close of the pilot. Guided by the Consolidated Framework for Implementation Research, a research associate queried CHWs for their perceptions of the successes and failures in protocol implementation, acceptance of CHWs within the clinical framework, and suggestions for improving the approach. Data from the CHW focus group were analyzed by 2 graduate-level researchers trained in qualitative data analysis. A rapid thematic approach was employed, in which each analyst independently summarized the focus group recording down to its key points and reviewed their summary for emerging themes. The analysts then met to compare their findings, discuss any edits, and finalize the list.

### Preliminary Efficacy Measures

Three survey instruments were examined to assess the ease of delivery and ability to capture patient experiences before and after the intervention. The measurements—collectively called the INSTACARE Health Survey (INSTACARE-HS)—combined 3 validated assessments based on the barriers and challenges previously identified in our formative research: the Medical Outcome Study HIV Health Survey (MOS-HIV), the Doctor-Patient Communication Questionnaire (DPC), and the Everyday Discrimination Scale (EDS).

The MOS-HIV is a 30-item assessment with 11 domains for quality of life [16]. MOS-HIV domains were scored by summing the item responses after recoding for reverse-scored items. The scales for each domain were then transformed to a scale from 0 to 100, with a higher score representing better health status [16]. The EDS is an 8-item survey on a 4-piont scale that assesses how often participants experienced mistreatment within the past year [17]. The DPC questionnaire is a 13-item survey also rated on a 4-point Likert scale that examines the relationship between how well a doctor communicates with their patients and the patient’s outcomes [18]. A low score on the EDS indicates low perceived discrimination, while a high score on the DPC suggests strong communication between patients and physicians. As with the MOS-HIV, scores for both assessments were averaged and transformed to a scale from 0 to 100. All 3 assessments were reviewed by key community and health system stakeholders prior to implementation.

### Data Analysis

The sociodemographic factors of the sample were characterized using descriptive statistics. Data were collected on paper forms and entered into a REDCap (Research Electronic Data Capture; Vanderbilt University) database by a member of the research team. Data were analyzed using SPSS (version 28; IBM Corp). Continuous variables were expressed using median and IQR, and categorical variables were expressed as frequencies.

## Results

### Participants

The 10 participants were evenly distributed by gender (ie, 5 women and 5 men), and their ages ranged from 26 to 64 years with a median age of 55 (IQR 46-58) years. Three (30%) participants (2 men and 1 woman) were of Haitian descent; the remaining (n=7, 70%) identified as African American or reported no ethnic identity. Baseline viral load levels ranged from 214 to 343,000 copies/mL. Half (n=5, 50%) of participants identified their greatest barrier to adherence as an unmet basic need, most commonly food and shelter. Three (30%) participants identified health care–related barriers, such as adverse medication side effects or identifying covered providers, whereas 2 (20%) indicated social barriers, such as a limited support network.

### Intervention Feasibility

Participant recruitment rate was projected to be 10 participants within 1 month (approximately 1 participant every 3 days) based on prior research by the investigative team [8]. Turnover in clinic staff and an institutional hiring freeze led to initial delays in recruitment from December 2022 to March 2023, resulting in 3 participants being enrolled via direct provider referral from December 2022 to February 2023. Due to the unusual administrative disruption in the recruitment strategy, these 3 cases were treated as outliers in the data. Thus, a more accurate recruitment rate was inferred from the remaining data.

**Figure 1:**
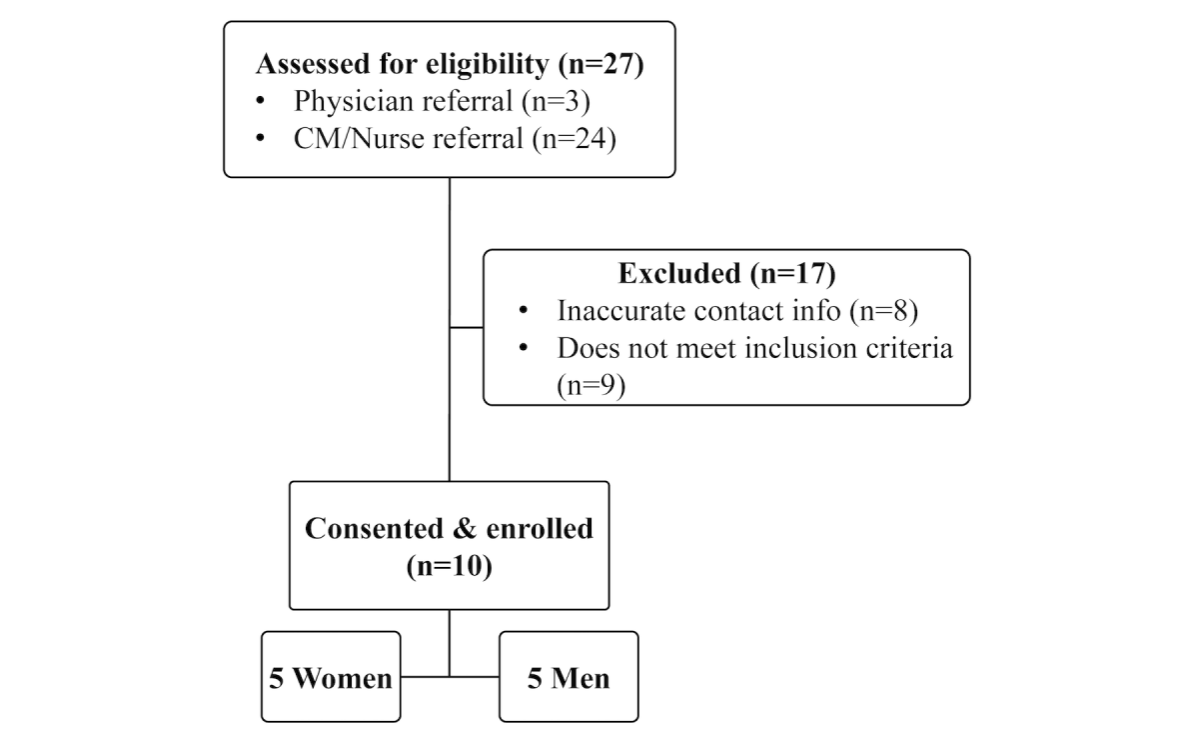
Flowchart depicting the enrollment process for participants in the INSTACARE pilot study in Miami, Florida. CM: case manager; INSTACARE: Integrated Navigation and Support for Treatment, Adherence, Counseling, and Research.

From April to May 2023, the remaining 7 participants were enrolled into the INSTACARE pilot. A total of 24 patients were identified as potential participants by the charge nurse and referred to the study team for screening before the target sample size was achieved. As shown in Figure 1, the 17 excluded potential participants were removed due to inaccurate contact information (n=9) or updated laboratory work indicating viral suppression (n=8). Thus, CHWs were able to recruit 7 participants within 5 weeks, resulting in an average recruitment of 1 participant every 5 days, approximately 60% of the projected recruitment rate. All eligible participants who were approached about the study agreed to participate.

Participant fidelity in the INSTACARE intervention was moderately strong in this small sample, with 7 (70%) of the 10 participants attending more than 75% of their CHW sessions. Notably, participants who were disengaged with the intervention became more involved within their last few weeks, and the majority (8/10, 80%) asked to participate if a larger intervention became available.

### Preliminary Efficacy

**Figure 2:**
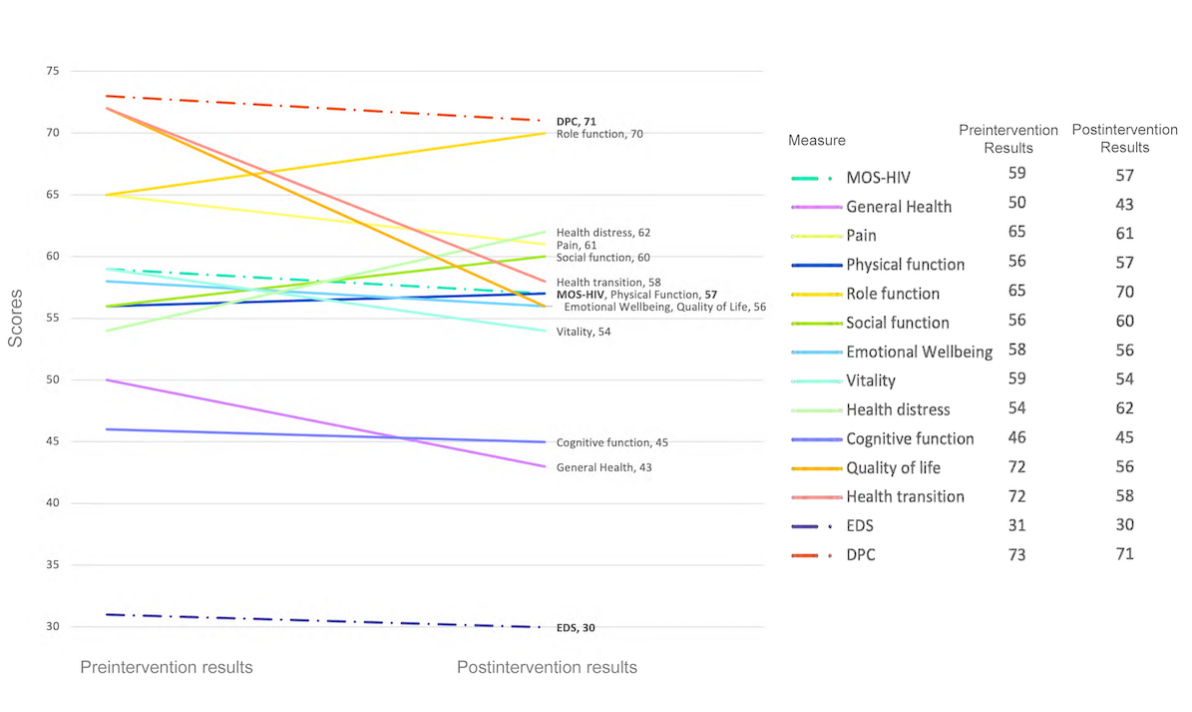
Average preintervention and postintervention results on the INSTACARE Health Survey among a sample of Black people living with HIV in Miami, Florida. DPC: Doctor-Patient Communication Questionnaire; EDS: Everyday Discrimination scale; INSTACARE: Integrated Navigation and Support for Treatment, Adherence, Counseling, and Research; MOS-HIV: Medical Outcome Study HIV Health Survey.

As a pilot, feasibility, prospective cohort study with a small, nonrandomized sample size, this study was not adequately powered to identify statistically significant differences between the baseline and exit assessments. Thus, the INSTACARE-HS instrument was evaluated on its efficacy as a data collection tool, and pre-post survey responses were reviewed for potential preliminary trends among survey responses. CHWs reported that the 55-question survey took, on average, 60 to 90 minutes to complete. As shown in Table 2, no considerable changes were observed between the baseline and exit scores for the EDS and DPC assessments. The average EDS score was 30 (SD 26.7), suggesting low to moderate levels of perceived discrimination among patients. The average DPC score was 72 (SD 26.4), indicating good communication between patients and providers. Some improvement was observed across multiple MOS-HIV domains between preintervention and postintervention results, including health distress, role limitation, and social functioning (Figure 2).

Changes in HIV viral load were also reviewed. At study exit, 4 (40%) of the 10 participants had achieved viral suppression within the 3-month study timeframe, and 1 (10%) experienced a clinically significant reduction in viral load, although they did not reach viral suppression. Of the 5 remaining participants, 4 did not complete their scheduled laboratory work, including 1 who refused ART treatment. Finally, 1 participant maintained high intervention compliance but experienced worsening HIV outcomes due to the advanced progression of their HIV and an excess of social and structural barriers, including homelessness, unemployment, limited literacy, and recent traumatic injury.

**Table 2:** INSTACARE^a^ Health Survey assessments, scoring, and average pre-post results among a sample of Black people living with HIV in Miami, Florida.

Assessment			Items, n		Score range		Preintervention score, mean (SD)		Postintervention score, mean (SD)
**Medical Outcome Survey HIV Health Survey**			30		0-100		59 (8.4)		57 (7.5)
	General health	1		1-5		50 (31.1)		43 (23.7)	
	Pain	2		2-15		65 (18.3)		61 (30.9)	
	Physical function	6		6-18		56 (29.1)		57 (34.2)	
	Role function	2		2-4		65 (41.2)		70 (42.1)	
	Social function	1		1-6		56 (37.5)		60 (35.2)	
	Emotional well-being	5		5-30		58 (19.4)		56 (26.8)	
	Vitality	4		4-24		59 (24.8)		54 (28.2)	
	Health distress	4		4-20		54 (33.9)		62 (36.1)	
	Cognitive function	3		4-24		46 (32.6)		45 (22.9)	
	Quality of life	1		1-5		72 (23.2)		56 (16.7)	
	Health transition	1		1-5		72 (29.2)		58 (37.5)	
**Everyday Discrimination Scale**			8		8-32^b^		31 (30.1)		30 (26.7)
**Doctor-Patient Communication Questionnaire**			13		13-52^b^		73 (33.6)		72 (26.4)

^a^INSTACARE: Integrated Navigation and Support for Treatment, Adherence, Counseling, and Research.

^b^Final scores are scaled to be out of 100.

### CHW Perspectives

Emergent themes from the focus group reflected CHW insights on the challenges surrounding study implementation and the acceptability of the intervention within the clinic environment. A theme reiterated throughout the group discussion was the need for more time with participants. While the CHWs believed they could help participants overcome their barriers to HIV treatment, they also felt that they were “just starting to peel the layers” for many of their difficult cases. This is further reflected in CHW case notes, which highlight how the participants missed fewer CHW check-ins leading up to their exit date.

CHWs further indicated that many of the resources that participants needed for adherence were social services that could not be addressed within the pilot timeframe. For example, some social services, such as housing or food assistance, require specific documentation that is often unavailable to the most vulnerable members of the population. This was the case for 1 houseless participant, who needed to replace their state ID and social security card to apply for disability benefits. Receipt of this documentation can take up to 2 weeks from the point of submission, and this paperwork must then be brought to the appropriate city office for processing. Further, these estimates do not account for the time potentially needed to locate a transient individual once the documentation arrives. One CHW shared the following:

Getting HIV under control is a priority, but it’s not the Number 1 priority...it’s housing or it’s some sort of income. So, it becomes difficult because of...the timeframe that it takes to get those resources. We[’re] not only working with the individual and their HIV – we’re working with the individual and their HIV and the a-million other things that come with that.

CHWs also noted variability in participant engagement with the intervention. Half (5/10, 50%) of the sample was actively engaged in consistent communication with their CHW, attending 100% of CHW sessions and freely opening up about their barriers to care. Others, however, were more hesitant to establish open dialogue, while some seemed to avoid CHW contact for the majority of the intervention. As one CHW described, “I felt like [the] FBI!” highlighting the challenges to locate some of her patients and provide the resources they discussed. Despite limited engagement, the CHWs noted that 9 (90%) of the 10 participants showed increased communication and engagement in the final weeks of the intervention and believed that they could develop a rapport with each patient in time. CHWs also highlighted the INSTACARE-HS as a beneficial rapport-building tool. As some clients were less forthright with their need for assistance compared to others, the baseline survey provided CHWs with valuable insight on the social barriers and experiences of the clients. All participants reported interest in continued CHW support if another study became available, suggesting that they were able to establish a connection with their CHW during the intervention timeframe. These findings are further supported throughout CHW-focused research, as several publications reflect that “trust cannot be earned overnight” [19].

CHWs held mixed views on their relationships with clinic staff. They perceived the nurses and physicians as having “bought-in” to the clinic-integrated CHW model, as many of them have participated in CHW interventions in the past. As a result of these prior experiences, one CHW stated the following:

They know the benefit of having a CHW...a one-on-one individual that helps [patients] not only navigate the healthcare system, but [also] to better understand and identify issues in their environment.

With regard to social services and support staff, however, CHWs felt that their role was met with “resistance,” largely due to misperceptions surrounding the role of a CHW within the clinical setting:

Individuals who render services to the same scope of patients that we target, they don’t necessarily see us as an ally – they see us as repetitive resources.

The group also believed that this barrier could be overcome with time as staff witnessed CHWs work in action and distinguished their services from those of traditional case management.

Our line of work is different from a regular case manager, so people don’t fully understand [because] they see us as case management.

## Discussion

This was a pilot, feasibility, prospective cohort study examining the feasibility of a clinic-integrated CHW intervention designed to help Black people living with HIV achieve viral suppression. Specifically, this study evaluated feasibility across four criteria: (1) an estimated recruitment rate of 10 participants per month; (2) a balanced sample composition recruited from the existing clinic population; (3) intervention fidelity among study participants; and (4) CHW perspectives on study implementation. The findings of this study suggest that clinic-integrated CHWs are a feasible and acceptable approach to facilitate greater access and communication between patients and the health system and, thus, may improve patient outcomes along the continuum of care.

Approximately half of the Black people living with HIV in Miami have fallen from HIV care. Despite increased use of antiretroviral medication and an overall increase in sustained viral suppression among the general population, fewer than 41% of Black adults have achieved viral suppression [20], highlighting the significant barrier that social determinants of health can create beyond access to care. This is further supported by our data, as the most common barriers to viral suppression for this sample were basic physiological needs, such as food and shelter. As a resource with an established presence in clinical and community settings, CHWs can help people living with HIV address these factors and prevent them from falling through the cracks in the health care system.

Aligned with the literature, results from this intervention highlight the importance of both internal and external stakeholder buy-in when establishing a clinic-focused CHW system [21]. While CHWs felt that they created a collaborative relationship with the majority of nurses and physicians, support from hospital CMs and social workers was difficult to obtain during pilot implementation, as many felt that the CHW role was a replacement for the services social workers currently provide. Future studies should prioritize the development of a research advisory board during the study design phase to ensure that the perspectives of all key stakeholders are included prior to implementation.

When considering generalizability, this study was focused on a small sample of patients at a single outpatient HIV clinic. As a result, these findings may not be generalizable to other settings or populations, such as individuals that are lost to care or in treatment at private facilities. However, as the largest public hospital in Miami-Dade County and the third largest safety-net hospital in the United States, this health center serves as a primary entry point to obtain HIV care among underserved minority populations. While the broader applicability of this sample is limited, patients seeking care at this institution are often those that lack the resources to obtain services elsewhere and, similarly, may reflect people living with HIV in Miami who would most benefit from CHW support.

Our assessment of CHW fidelity to the intervention was also limited in its results. While all CHWs were trained in HIV counseling, research file management, and human subjects research strategies, our assessment of CHW fidelity to intervention delivery would have benefit from additional quality control procedures, such as randomized files reviews and client audits. Case notes and other documentation on participant interactions were handwritten by CHWs and reviewed by the research coordinator for completion. However, verification of these interactions would have strengthened our evaluation of CHW interventions and limited the influence of potential moderators. As described by the Conceptual Framework for Implementation Fidelity [22], complex interventions, such as sociobehavioral studies, have greater scope for variation in their delivery and, thus, are more susceptible to deviations from the protocol. As such, more rigorous recording, reporting, and verification of implementation delivery are needed to ensure the accuracy and replicability of complex intervention methods, and such strategies should be incorporated into future efficacy studies.

Finally, the study activities involved in this CHW intervention are designed for a 1-year implementation period. Our previous research on CHW strategies for treatment adherence among Black people living with HIV found that intervention delivery must last at least 24 weeks to achieve significant improvements in adherence. While this study was not powered to identify statistically significant differences in patient outcomes, many of our survey domains were trending in a positive direction despite the limited timeline, and greater improvements may have been observed if the intervention was implemented for longer. To better determine the efficacy of this intervention, a fully powered randomized trial is necessary.

As an often underutilized resource in the health care system, CHWs are a valuable asset to health care teams due to their unique bond with the population of interest. Our study demonstrates that a clinic-embedded CHW intervention is a feasible strategy with the potential to improve treatment adherence by addressing adverse social determinants of health. By offering targeted social and clinical support, this research shows that CHWs may be a promising solution to achieve sustained viral suppression and care engagement for Black people living with HIV.

## Abbreviations

ART: antiretroviral therapy
CHW: community health worker
CM: case manager
DPC: Doctor-Patient Communication Questionnaire
EDS: Everyday Discrimination Scale
INSTACARE: Integrated Navigation and Support for Treatment, Adherence, Counseling, and Research
INSTACARE-HS: Integrated Navigation and Support for Treatment, Adherence, Counseling, and Research Health Survey
MOS-HIV: Medical Outcome Study HIV Health Survey
REDCap: Research Electronic Data Capture
